# Dissociations and interactions between time, numerosity and space processing

**DOI:** 10.1016/j.neuropsychologia.2009.05.024

**Published:** 2009-11

**Authors:** Marinella Cappelletti, Elliot D. Freeman, Lisa Cipolotti

**Affiliations:** a Institute of Cognitive Neuroscience, University College London, 17 Queen Square, London WC1N 3AR, UK; b Department of Neuropsychology, National Hospital for Neurology and Neurosurgery, Queen Square, London WC1N 3BG, UK; c Department of Psychology, University of Palermo, Via Delle Scienze 15, Palermo, Italy

**Keywords:** Time, Magnitude, Numerosity, Space, Number cognition

## Abstract

This study investigated time, numerosity and space processing in a patient (CB) with a right hemisphere lesion. We tested whether these magnitude dimensions share a common magnitude system or whether they are processed by dimension-specific magnitude systems. Five experimental tasks were used: Tasks 1–3 assessed time and numerosity independently and time and numerosity jointly. Tasks 4 and 5 investigated space processing independently and space and numbers jointly. Patient CB was impaired at estimating time and at discriminating between temporal intervals, his errors being underestimations. In contrast, his ability to process numbers and space was normal. A unidirectional interaction between numbers and time was found in both the patient and the control subjects. Strikingly, small numbers were perceived as lasting shorter and large numbers as lasting longer. In contrast, number processing was not affected by time, i.e. short durations did not result in perceiving fewer numbers and long durations in perceiving more numbers. Numbers and space also interacted, with small numbers answered faster when presented on the left side of space, and the reverse for large numbers. Our results demonstrate that time processing can be selectively impaired. This suggests that mechanisms specific for time processing may be partially independent from those involved in processing numbers and space. However, the interaction between numbers and time and between numbers and space also suggests that although independent, there maybe some overlap between time, numbers and space. These data suggest a partly shared mechanism between time, numbers and space which may be involved in magnitude processing or may be recruited to perform cognitive operations on magnitude dimensions.

## 1 Introduction

The issue of whether the human brain processes various types of magnitude through a shared mechanism or whether we have different magnitude representations for each magnitude dimension has been the focus of recent behavioural, animal and neuroimaging research. Neuropsychological evidence so far has been rather scarce.

### 1.1 One common magnitude system

It has been suggested that a single fully shared representational mechanism may underpin time and numerosity processing (Meck & Church, 1983). Based on evidence coming from animal studies, this single mechanism has been hypothesized as an ‘internal accumulator’ which represents the duration or the numerosity of events/objects (Boysen & Capaldi, 1993; Breukelaar & Dalrymple-Alford, 1998; Meck & Church, 1983; Roberts & Church, 1978). The internal accumulator is thought to sum up the impulses produced by a generator. For time perception this generator produces impulses each time an event is encountered, for numerosity each time an object is encountered (Meck & Church, 1983). Meck and Church (1983) also suggested that the accumulator can only receive information either about time or numerosity at one given time. Some authors have adopted a more radical interpretation of Meck and Church's view, and have suggested that time and numbers are also represented in the same format as continuous quantities (Gallistel & Gelman, 2004). From this perspective, two predictions can be made. First, no neuropsychological dissociations should be expected between time and numbers as impairments to the accumulator should equally affect both dimensions. Second, no interactions between these dimensions should be expected, as the accumulator is assumed to process time and numerosity one at a time.

The idea of a single representational mechanism supporting magnitude processing has been recently extended within a new theory termed ATOM (A Theory Of Magnitude, Walsh, 2003). According to this theoretical framework a single representational mechanism underpins time, quantity and also space. The ATOM proposes that these magnitude dimensions share an innate, common metric system for action which operates on a shared internal accumulator. Moreover, the common magnitude system is only partly shared among magnitude dimensions as each of them is also implemented by dimension-specific processes (Walsh, 2003, Fig. 1b). The ATOM differs from previous accounts as it includes ‘space’ as an additional magnitude dimension, it assumes a partly and not fully shared accumulator mechanism, and it hypothesises that this common magnitude system is located in the right parietal lobe. However, at present the ATOM is underspecified and makes no clear predictions about the relation between the different magnitude dimensions and about the type of cognitive mechanisms and neural correlates which are shared or distinct for each magnitude dimensions.

### 1.2 Multiple magnitude systems

An alternative to the hypothesis of common magnitude system is that magnitude dimensions are distinct and that there are multiple magnitude-specific systems. Although not systematically formulated, this possibility is mentioned by some authors (e.g. Moyer & Landauer, 1967; Walsh, 2003). This hypothesis predicts that magnitude information is analysed separately for time, numbers and space and compared according to metrics unique to each comparison. It has been suggested that indirect support to this hypothesis may come from some imaging studies reporting greater activation for numbers or for countable stimuli compared to other continuous magnitude dimensions such as luminosity or size (e.g. Castelli, Glaser, & Butterworth, 2006; Fias, Fize, Georgieva, & Lammertyn, 2003; Pinel, Piazza, Le Bihan, & Dehaene, 2004; but see Cohen Kadosh, Lammertyn, & Izard, 2008). Yet, greater brain activations for numbers would not necessarily correspond to distinct cognitive processes involved in time, number and space processing. For instance, some authors have suggested that higher activation for numbers may be due to more cognitive resources needed for numerical compared to analogue processing of physical size (e.g. Zorzi & Butterworth, 1999). Other authors, however, have suggested that there is a shared neuronal substrate for numbers and space, but not for other types of magnitude that are non-spatial (Pinel et al., 2004). Two predictions follow from the hypothesis of a distinct magnitude dimensions. First, dissociations between magnitude dimensions are possible as they are thought to be independent. Second, no interactions should occur between magnitude dimensions as the magnitude information is processed independently.

A variant of this proposal would suggest that magnitude dimensions can be distinct although they share the same operational mechanisms, for instance comparison mechanisms (Cohen Kadosh et al., 2008). Support to this hypothesis comes from studies that looked at whether processing numbers and other magnitude dimensions activate same or different responses. These studies hypothesized that if operational mechanisms such as those involved in comparison are shared between different magnitude dimensions, then interactions between dimensions are expected. A recent study combining fMRI and ERP showed that numbers and physical size modulated activity in the intraparietal sulcus (IPS), suggesting that their magnitude was processed through a common mechanism (Cohen Kadosh et al., 2007). In addition, these dimensions also interacted in the primary motor cortex, which was interpreted as evidence that numerical and size value were processed separately until response-related stages (Cohen Kadosh et al., 2007). At present it is unclear whether time, number and space processing are distinct but share operational mechanisms. Should this be the case, it is possible to predict that dissociations between dimensions may occur as well as interactions between them.

### 1.3 Interactions between magnitude dimensions

One way to test whether there are one or more magnitude systems is by looking for possible interactions between magnitude dimensions. The prediction is that no interactions should occur if these dimensions are thought to be independent from each other and implemented by independent magnitude systems. Moreover, no interactions should occur on the basis of a fully shared magnitude system where information about various magnitude dimensions can only be processed for one dimension at a time. On the other hand, both the hypotheses of a partly shared mechanism between these dimensions or of shared operational mechanisms allow us to expect interactions between them. These interactions can be symmetric or bidirectional if two dimensions interfere with or facilitate each other, such that, for example, processing time is influenced by numerosity and processing numerosity is influenced by time. Interaction between magnitude dimensions can also be unidirectional such that, for example, only numerosity would influence time or vice versa. It has also been suggested that the asymmetric interference or facilitation of a dimension over another may indicate that this dimension is more salient or that it is processed faster or more efficiently (e.g. Dormal, Seron, & Pesenti, 2006).

Behavioural studies exploring interactions between *time and numerosity* have so far reported unidirectional interactions between these dimensions, with time processing more often affected by numerosity processing (e.g. Dormal et al., 2006; Dormal, Andres, & Pesenti, 2008; Droit-Volet, 2003). For instance, many studies have shown that when subjects perform a numerical task or judge the numerosity of non-symbolic quantity stimuli such as dots, temporal intervals are perceived as shorter than their veridical duration (e.g. Burnside, 1971; Dormal et al., 2006, 2008; Droit-Volet, 2003; Gulliksen, 1927; Hawkes & Sherman, 1972; Xuan, Zhang, He, & Chen, 2007). These unilateral interactions of numbers over time have sometimes been explained in terms of their automatic access, namely numbers would modulate performance even when irrelevant for the task (e.g. Dormal et al., 2006). So far, only one study has reported an effect in the opposite direction, whereby time processing interfered with efficient mental arithmetic. This interference of arithmetic on time perception has been interpreted as involving more generalized dual-task effects (Brown, 1997).

Evidence for bidirectional interaction between *space and numbers* comes from studies showing that space affects number processing (i.e. SNARC effect, spatial numerical association of response codes, Dehaene, Bossini, & Giraux, 1993) as well as that numbers can influence performance on spatial cognitive tasks (e.g. Bonato, Priftis, Marenzi, & Zorzi, 2008, Calabria & Rossetti, 2005; de Hevia, Girelli, & Vallar, 2006; Fischer, Castel, Dodd, & Pratt, 2003; Hubbard, Piazza, Pinel, & Dehaene, 2005; Kaufmann et al., 2005; Stoianov, Kramer, Umiltà, & Zorzi, 2007).

### 1.4 The anatomical basis of time, numbers and space

Neuroimaging studies have mainly explored time, numbers and space independently and have consistently reported activations of the parietal regions among others (e.g. for time: Ivry, 1996; Lejeune, El Ahmadi, & Weyers, 1997; Maquet et al., 1996; Rao, Mayer, & Harrington, 2001; Schubotz, Friederici, & Yves von Cramon, 2000; for numbers: Cappelletti, Lee, Freeman, & Price, in press; Dehaene, Piazza, Pinel, & Cohen, 2003; Pesenti, Thioux, Seron, & De Volder, 2000; Pinel et al., 2004; for space: Medendorp, Goltz, Vilis, & Crawford, 2003; Merriam, Genovese, & Colby, 2003). Moreover, some studies investigating time and numbers together have shown that the activation in the right inferior parietal lobe (Talairach co-ordinates 44 −52 40, Pouthas et al., 2000) cancelled out when the two conditions were subtracted from each other, thus providing indirect evidence that these dimensions may rely on similar brain areas (e.g. Maquet et al., 1996; Pouthas et al., 2000).

This evidence would be consistent with the ATOMs proposal that a precise neurological basis for the common magnitude system is located in the right parietal regions (Walsh, 2003). Nevertheless, the findings that time, numbers and space independently engage the parietal areas cannot be taken as conclusive evidence that they share a common magnitude system because a common brain region may underpin different cognitive mechanisms. In addition, the parietal lobes have been shown to be recruited in several conceptual, perceptual and perceptual-motor transformations such as response-selection processes which may be in common to time, number, and space processing (e.g. Bunge, Hazeltine, Scanlon, Rosen, & Gabrieli, 2002; Corbetta & Shulman, 2002; Culham & Kanwisher, 2001; Richter et al., 2000; Wojciulik & Kanwisher, 1999). That is, extracting and comparing learnt information from stimuli or selecting a response such as a left or right key press might engage the same parietal areas irrespective of the cognitive task performed. Therefore similar parietal activations during temporal, numerical and spatial tasks may not necessarily be evidence of a shared magnitude system between these dimensions. For example, a recent study using transcranial magnetic stimulation (TMS) indicated that the parietal areas critical for time estimation are distinct from those involved in numerosity or quantity processing (Dormal et al., 2008). Specifically, numerosity but not time estimation was impaired only following stimulation over the left IPS. Stimulation over the right IPS did not result in any interference for either time or numerosity estimation, suggesting that distinct cerebral locations are responsible for processing time and numerosity (Dormal et al., 2008). Similarly, Alexander, Cowey, and Walsh (2005) showed that TMS on the right posterior parietal lobe impairs (i.e. increases reaction times) in a time estimation task but not in a quantity task (pitch comparison, Alexander et al., 2005).

### 1.5 The neuropsychology of time, numbers and space

As far as we are aware, there has been no neuropsychological research that has directly investigated time, numbers and space within the same study. However, a few studies have focused on one or two of these dimensions and have reported selective impairments of time, numbers or space processing (e.g. time: Harrington, Lee, Boyd, Rapcsak, & Knight, 2004; Koch, Oliveri, Torriero, & Caltagirone, 2003; Meck, 2005; numbers: Cipolotti, Butterworth, & Denes, 1991; Lemer, Dehaene, Spelke, & Cohen, 2003; space: Driver & Vuilleumier, 2001). For instance, studies investigating space and numbers in patients with neglect showed similar impairment in processing the left hand side of physical lines and of mental number lines (Zorzi, Priftis, & Umiltà, 2002 but see Doricchi, Guariglia, Gasparini, & Tomaiuolo, 2005 for a different pattern of results). This association of spatial and numerical impairment seems to partially depend on the spatial orientation of the physical and number lines (Cappelletti, Freeman, & Cipolotti, 2007). Lesion studies exploring time, number and space processing within the same study are clearly needed to test whether these dimensions are implemented by independent magnitude systems or by a common one.

This study reports for the first time a patient with a selective impairment in time processing following a right hemisphere lesion. We tested: (1) whether number and space processing were also impaired or spared; (2) whether there was any interaction between these dimensions, and the nature of any such interaction, i.e. unidirectional or bidirectional. Our reasoning was as follows: (i) if there is a fully shared magnitude system between time, numbers and space, then impairment in one of these three magnitude dimensions should coincide with impairments in the other two dimensions; (ii) conversely, if time, numbers and space processes are independent, then it should be possible to reveal selective impairments in some of them; selective impairments may also be predicted following the hypothesis that there are dimension-specific processes as well as a partly shared common magnitude system; (iii) interactions between magnitude dimensions are predicted based on the idea of a partly shared common magnitude system or operational mechanisms. Our experiments tested for any interaction between the magnitude dimensions we studied, i.e. of numbers on time and space, of time on numbers, and of space on numbers.

## 2 Participants

### 2.1 Patient CB

#### 2.1.1 Case history

CB is a 62-year-old right-handed native English-speaking man with university education. In 2004 he sustained a right middle cerebral artery territory infarct. The present research was conducted in 2007.

#### 2.1.2 MRI findings

**Fig. 1:**
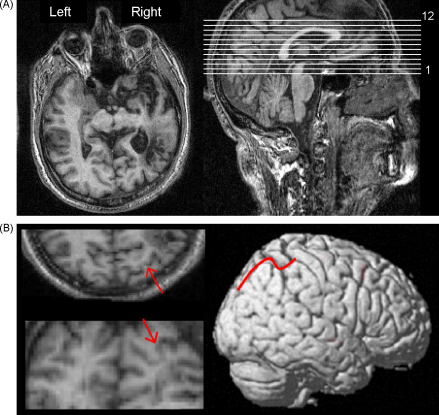
(A) Patient CBs brain scan in the axial plane (left) in relation to a template (right). (B) The patient's intraparietal sulcus indicated by red arrows in the axial (top left) and coronal (bottom left) views in relation to a template (right).

An MRI-scan showed an extensive right hemisphere lesion involving the right inferior parietal regions extending to the right superior temporal lobe. Damage was also shown in the right inferior frontal and lateral prefrontal areas around the Silvian fissure extending deeply into the insula and the right basal ganglia. The intraparietal sulcus (IPS) was intact and the cerebellum was normal (see Fig. 1A and B and Appendices A and B). In order to measure the volume occupied by the patient's lesion relative to the whole brain, CBs lesion was plotted from the clinical MR scan onto a standard CH2 template using MRIcro software (www.mricro.com). After conversion of regions of interest (ROIs) to voxels of interest (VOIs), the volume of the patient's lesion and of the whole brain was estimated using MIPAV software (Centre for Information Technology, Bethesda, MD). Using this technique, it was calculated that the patient's lesion occupied 19.8% of the whole brain volume.

#### 2.1.3 Neuropsychological examination

The patient was referred to the Neuropsychology Department of the National Hospital for Neurology and Neurosurgery in London for the evaluation of his cognitive impairments. The neuropsychological assessment took place around the time of the experimental investigation. The results are reported in Table 1.

On the WAIS-R (Wechsler, 1981), the patient obtained a high average verbal I.Q. and average performance I.Q. suggesting a mild degree of intellectual underfunctioning only on test with a non-verbal component. Visual and verbal memory functions were normal. Similarly, nominal and frontal executive functions were also entirely normal. Two subtests from the Test of Everyday Attention (Robertson, Ward, Ridgeway, & Nimmo-Smith, 1996) were administered to assess our patient's attention. His performance was normal both on the ‘elevator counting’ and on the ‘elevator counting with distractors’. The patient performed flawlessly on three subtests from the VOSP (Warrington & James, 1991), and he obtained a score in the 10 grade on the Graded Difficulty Arithmetic Test (Jackson & Warrington, 1986), corresponding to average performance. Moreover, there was no evidence of neglect as his performance on three tests sensitive to neglect was intact. In summary, the patient had only a mild degree of intellectual underfunctioning on the non-verbal part of the WAIS-R.

Nevertheless, the patient reported a number of difficulties in everyday life related to processing time. He complained of not being able to make plans involving time. For example he was very bad at deciding when it was the right time to leave home to be on time for an appointment. Similarly, he often grossly misjudged the amount of time that was needed to come back and for everyday activities such as for example shopping and personal care. As a result of this, his daily routine became somewhat erratic. Examination of time processing based on paper-and-pencil tasks was first administered to evaluate CBs time processing abilities. Moreover, since difficulties in processing time may be related to numerical impairments we administered a series of numeracy and quantity processing tests (see Section 2.3).

### 2.2 Control subjects

Overall 18 neurologically healthy control subjects participated in the study (8 males, mean age: 63.8 years, SD 3.2). They were matched to the patient for age and I.Q. The patient and all control subjects gave informed written consent to participate in the study in accordance with the local research ethics committee. They performed all paper-and-pencil and computer-based tasks. Specifically, 5 subjects performed Task 5 (time discrimination), 8 subjects performed Tasks 1 and 3 (time estimation) and Tasks 6–8 (space and space and numbers), 12 subjects (4 new) performed Tasks 2 and 4 (numerosity estimation). In total, six control subjects took part in all experimental tasks, with the exception of Task 5 which was modified from an existing paradigm (Oliveri et al., 2008).

### 2.3 Preliminary investigation: time, numeracy and space processing

Three paper-and-pencil tasks were used to assess time. The ‘time estimation’ task required participants to estimate the time needed to perform familiar actions (e.g. making a cup of tea) or the duration of events (e.g. flying from London to New York). The ‘knowledge of exact temporal facts’ task required participants to answer questions about exact temporal facts (e.g. how many hours in a day?). The ‘time comparison’ task required participants to indicate the later time among two (e.g. ‘22.30’ vs ‘22.53’).

Numeracy processing was assessed with eight tasks, five tasks requiring estimation of numbers, areas and calculation, and three requiring exact numeracy processing. Numerosity estimation was assessed with the following tasks: (1) Numerical estimation task, requiring participants to estimate the size of objects (e.g. the area of a tennis court) or the number of items in a set (e.g. the number of eyelashes on one eyelid). (2) Number comparison estimation, where participants were asked to select the target number closer in magnitude to a 1-to-4 digit reference number. (3) Calculation estimation, requiring participants to approximate the result of arithmetical operations without calculating it. (4) Area estimation, requiring participants to estimate and compare, in two different tasks, the area covered by two sets of square figures. (5) Number of squares estimation, requiring participants to estimate and compare, in two different tasks, the number of squares contained in two rectangular figures.

Exact numeracy processing was assessed with three tasks: (1) Knowledge of exact number facts, requiring to answer questions tapping knowledge of exact number facts (e.g. how many strings are on the guitar?). (2) Knowledge of arithmetical facts requiring participants to answer questions such as ‘3 × 9’ or ‘7 + 8’. (3) Number comparison requiring subjects to indicate the larger between two numbers.

Space processing was assessed with general background tasks (see above). In addition, we used a ‘Location Detection’ task where participants had to identify the position of dots on the monitor. A single black dot (1.8° of visual angle) was randomly presented in one of four quadrants of the computer monitor (upper left and right, lower left and right) with an eccentricity of 8° of visual angle relative to the central fixation point. Each trial consisted of a fixation point presented in the middle of the monitor for 200 ms, followed by a single dot for 100 ms. Stimuli were presented on the four quadrants with equal frequency in pseudo-random order over two blocks of 120 stimuli each. Participants had to verbally report whether each dot was presented on the upper left or right, lower left or right quadrant of the monitor. Answers were recorded and scored by the experimenter.

Correct answers were given a score of 1; in tasks requiring an estimate, correct answers were within 2 standard deviations from controls. Relative to control subjects patient CB was impaired at ‘time estimation’ task as he misjudged the duration of familiar events or the time required to perform actions (based on the significance test (ST) of Crawford & Garthwaite, 2002; Crawford, Howell, & Garthwaite, 1998), ST: *t*(9) = 4.6, *p* < 0.001, see Table 2A.^1^ In contrast, his ‘knowledge of exact temporal facts’ and ‘time comparison’ were preserved (no difference with control subjects, ST: *t*(9) = 0.25, *p* *<* 0.8 and ST: *t*(9) = 0.23, *p* *=* 0.82, respectively, see Table 2B). Relative to control subjects, the patient's numerosity estimation and exact numeracy processing were both preserved (ST: *t*(9) = 0.15, *p* < 0.4 and ST: *t*(9) = 0.005, *p* < 0.1, respectively). Control subjects performed well in all paper-and-pencil tasks assessing time and numeracy skills. In the Location Detection Task, patient CB correctly indicated the position of stimuli on the monitor (113 out of 120 correct answers > 0.25 guessing rate, Binominal probability *p* *<* 0.001). Similarly, control subjects showed no impairment in the Location Detection Task (115 out of 120 correct answers > 0.25 guessing rate, Binominal probability *p* *<* 0.001). There was no significant difference between patient CB and control subjects’ performance in this task (ST: *t*(9) = 0.615, *p* = 0.56, n.s.).

### 2.4 Interim discussion

These results suggest that patient CB was impaired in processing time. In contrast, the examination of his numeracy skills and spatial functioning showed that they were intact. Therefore, it seems that in patient CB time was selectively impaired, while number and space were selectively spared. In order to provide a more specific account of this possible selective impairment, the experimental investigation explored CBs’ time, number and space processing with a set of computerized tests. It is possible that the paper-and-pencil tasks and the computerized tasks may be tapping into different types of timing computations, for example in terms of supra-second and sub-second intervals which may in turn involve different systems (e.g. Ivry & Spencer, 2004; Matelli & Meck, 2000, 2004; Meck & Benson, 2002). However, our aim was to evaluate CBs ability to process time irrespective of the time range.

## 3 Experimental investigation

There were five experimental sections. In sections one to three, five tasks were used to assess time and number processing individually (Tasks 1, 2 and 4), and time and numbers jointly (Tasks 3 and 5). In sections four and five, three tasks were used to assess space processing individually (Task 6), and space and numbers jointly (Tasks 7 and 8). See Appendix C for a schematic representation of the design of the experimental tasks used. Space and time jointly could not be tested because of time constraints. All tasks in the five sections were administered to patient CB and to control subjects in separate testing sessions. In all tasks, participants sat in a quiet room facing the computer screen with their head on a chinrest. The viewing distance from the computer monitor was 50 cm. Stimulus presentation and data collection were controlled using the Cogent Graphics toolbox (http://www.vislab.ucl.ac.uk/Cogent/) and Matlab7 software on a S2VP Sony laptop computer. The dimensions of the display, as rendered on the built-in liquid-crystal screen, were 23.5 cm horizontal × 18 cm vertical. The display had a resolution of 640 × 480 pixels and was refreshed at a frequency of 60 Hz. When oral responses were made, these were recorded and scored by the experimenter. As we were interested in the participants’ response accuracy rather than their speed, un-timed oral answers were required in Tasks 1–4 and reaction times were not recorded in these tasks. Prior to the beginning of each task, 10 initial trials were given to the participants for training purposes. These trials were not included in analysis.

## 4 Data analysis

In Tasks 1–4 participants’ accuracy in time and numerosity tasks was assessed in the following way. First, we used linear regression to estimate the slope relating veridical to estimated time and numerosity judgements. If estimates were veridical, the value of this slope should be unity (1), while over- or underestimations should result in values larger or smaller than unity respectively. Second, to assess whether the slopes obtained from the control subjects were significantly different from unity, we constructed within-subjects 95% confidence intervals (Cousineau, 2005) based on the standard deviation of the slope estimate. Confidence intervals that included 1 would indicate no significant deviation from the prediction of veridical estimation. This confidence interval was also used to assess whether CBs performance lay within the normal range using Crawford et al.’s method (Crawford & Garthwaite, 2002, 2005; Crawford et al., 1998). In Tasks 1–4 we also tested the possible interactions between time and numbers, i.e. any effect of the duration and the size of the individual stimuli (only numerical stimuli for size) on estimates of duration and numerosity.

For the Time Discrimination task (Task 5), a similar method of psychometric function fitting as above was used to derive the test duration that was perceptually equivalent to the reference duration (i.e. the point of subjective equivalence, PSE).

For the Line Length Discrimination Task (Task 6), analysis proceeded by first plotting the psychometric function relating the probability of ‘test longer’ responses against the actual test line length. This function should typically increase from 0% to 100%, with 50% found where test length is perceptually equal to the reference line length (i.e. the point of subjective equivalence, PSE). A logistic function was then fitted to the data, using a least-squares algorithm (c.f. Wichmann & Hill, 2001). The aim of this procedure was to obtain the PSE and also the Just Noticeable Difference (JND) for each participant. The JND measures the minimal difference in length between test and reference lines that can be discriminated with reliable accuracy. The JND was computed by reading off from the fitted psychometric function the line lengths at which 25% and 75% of the responses were ‘test longer’, then dividing the difference between these line lengths by two (Coren, Ward, & Enns, 1999). To establish the extent to which CBs performance differed from the control subjects, CBs JND was expressed as a *z*-score relative to the controls’ JNDs.

A one-tailed significance test (ST) was used to compare the patient with the control group (Crawford & Garthwaite, 2002; Crawford et al., 1998). This test treats an individual patient as a sample, affording the comparison of the patient with the control group. One-tailed revised standardised difference test (RSDT) was used to test whether the discrepancy between scores on two tasks observed for a patient is significantly different from the discrepancies in the control sample (Crawford & Garthwaite, 2005; Crawford, Gray, & Garthwaite, 2003). The ‘SingSlope’ program (Crawford & Garthwhaite, 2005) was also used to compare the slope of the regression line of the patient with that of the control group. For all the tasks administered, the patient's performance was compared with that of all controls. Additionally, given that a different number of participants was used in various tasks, CBs performance was also compared to the subgroup of controls that performed all the experimental tasks.

Other standard non-parametric (e.g. Kruskal–Wallis) and parametric statistical tests (e.g. ANOVA, *t*-test) were also used to analyze results from the patient and the control sample, respectively.

### 4.1 Section 1. Time processing^2^

A ‘time estimation’ task (Task 1) was used where participants were required to estimate the duration of a set of stimuli. Stimuli consisted of circles with a diameter of 1.72° of visual angle, presented one at the time in the middle of the screen. The circles appeared in one or eight pre-selected colours (white, pink, red, green, yellow, grey, brown and blue) on a mid-grey background of luminance 44 cd/m^2^.

**Fig. 2:**
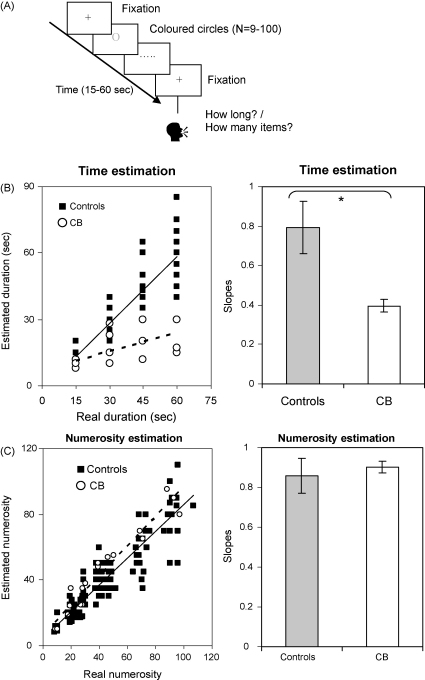
Tasks 1 and 2: Time and numerosity processing tested individually. (A) Design of Tasks 1 and 2; (B) performance of patient CB and control subjects in time and (C) numerosity estimation of non-numerical stimuli (coloured circles). Estimated durations (in s) and numerosities (number of items) are expressed as a function of real duration and numerosity respectively (left panels) and as slopes (right panels) with 95% confidence limits for patient CB and control subjects.

Each trial started with a central white cross that remained in the middle of the screen until subjects pressed the spacebar. Stimuli were presented one at a time in the central position until the selected time interval was completed. The number of stimuli ranged from 9 to 100. The end of a trial was indicated by the presentation of another white cross in the middle of the screen (Fig. 2A). The total duration of the sequence of stimuli was varied randomly over successive trials across four durations: 15, 30, 45 and 60 s. For data analysis, these sequence durations were grouped into two categories: short (15 and 30 s) and long (45 and 60 s). In successive trials, individual stimulus presentation times were sampled randomly from one of two continuous ranges: fast (200–1100 ms) and slow (1101–2000 ms). Each combination of stimulus duration (slow vs fast) and trial duration (short vs long) was sampled with equal frequency in 2 blocks of 16 trials each. At the end of each trial, participants were asked to estimate its duration in seconds or minutes and to verbally report their answer to the experimenter. There was no time constraint to produce an answer.

In order to prevent sub-vocal counting and to avoid strategies used to keep track of elapsing seconds, participants were required to name aloud the colour of each circle in each trial, following a procedure used in previous studies (e.g. Logie & Baddeley, 1987). Moreover, the fast presentation of the stimuli (i.e. 200–1100 ms for half of the trials) was designed to further prevent any sub-vocal counting.

#### 4.1.1 Results: time processing

In Task 1, patient CB consistently underestimated the duration of temporal intervals when coloured circles were used while performing a secondary task, i.e. colour naming (slope = 0.42, Standard Error (SE) = 0.045, significantly different from 1, *p* < 0.05, Fig. 2B). Control subjects showed no impairment in time estimation with these stimuli (slope = 0.955, SE = 0.08, not significantly different from 1, Fig. 2B). CBs performance was significantly different from control subjects [Satterthwaite's test (SingSlope, Crawford & Howell's test (Test a)): *t*(2) = −3.4, *p* < 0.03], even when compared to the subgroup of controls that performed all experimental tasks [Satterthwaite's test (SingSlope, Crawford & Howell's test (Test d.1)): *t*(2) = −6.9, *p* < 0.001].

#### 4.1.2 Interaction of stimulus duration on time estimation

An analysis was run to test whether performance was influenced by the duration of the individual stimuli. This showed that CBs time estimation was not influenced by the duration of the individual stimuli [Kruskal–Wallis main effect of stimulus duration, *χ*^2^ = 1.09, *p = *0.2, n.s.]. Thus the perceived duration of the whole sequence of events was neither shorter with short event durations (slope = 0.20, SE = 0.05), nor longer with long event durations (slope = 0.23, SE = 0.038).

Similarly, control subjects’ time estimation was not influenced by the duration of the individual stimuli [*F*(1, 8) = 0.11, *p* *=* 0.74, n.s.]. The effect of stimulus duration on time estimation did not differ between CB and the control group for both short [Satterthwaite's test (SingSlope, Crawford & Howell's test (Test d.1)): *t*(31) = −0.68, *p* = 0.49, n.s.] and long durations [Satterthwaite's test (SingSlope, Crawford & Howell's test (Test d.1)): *t*(31) = −0.7, *p* = 0.5, n.s]. This was also the case when CBs performance was compared to the subgroup of controls that performed all experimental tasks [short durations: SingSlope, Crawford & Howell's test (Test c): *t*(5) = −1.23, *p* < 0.22, n.s.; long durations: SingSlope, Crawford & Howell's test (Test c): *t*(5) = −4.8, *p* < 0.4, n.s.].

### 4.2 Section 2. Numerosity processing

A Numerosity Estimation task (Task 2) was used where participants were required to estimate the numerosity of a set of stimuli.^3^ The stimuli and experimental design of this task were identical to those used for the ‘time estimation’ task. The circles were presented in 2 blocks of 16 trials each. As previously, counting strategies were prevented by asking subjects to name the colour of each circle. There was no time constraint to produce an answer.

#### 4.2.1 Results: numerosity processing

Patient CB could correctly estimate the number of circles presented [slope = 1.01, SE = 0.03, not significantly different from 1, Fig. 2C]. Similarly, control subjects showed no impairment in numerosity estimation with these stimuli [slope = 0.9, SE = 0.01 not significantly different from 1]. There was no significant difference between patient CB and control subjects’ performance in numerosity estimation [Satterthwaite's test (SingSlope, Crawford & Howell's test (Test a)): *t*(14) = 0.29, *p* = 0.39, n.s.], even when CBs performance was directly compared to the subgroup of controls that performed all experimental tasks [SingSlope, Crawford & Howell's test (Test c): *t*(5) = 0.46, *p* < 0.66, n.s.].

A significant difference between time and numerosity estimation with non-numerical stimuli was found in patient CB relative to control subjects [RSDT: *t*(11) = 3.02, *p* < 0.04], such that only time but not numerosity processing was impaired. This difference was significant even when CBs performance was directly compared to the subgroup of controls that performed all experimental tasks [RSDT: *t*(5) = 4.41, *p* *=* 0.006]. The patient's pattern of performance therefore fulfilled the criteria for a classical dissociation (Crawford & Garthwaite, 2005; Crawford et al., 2003).

### 4.3 Section 3. Time and numbers

**Fig. 3:**
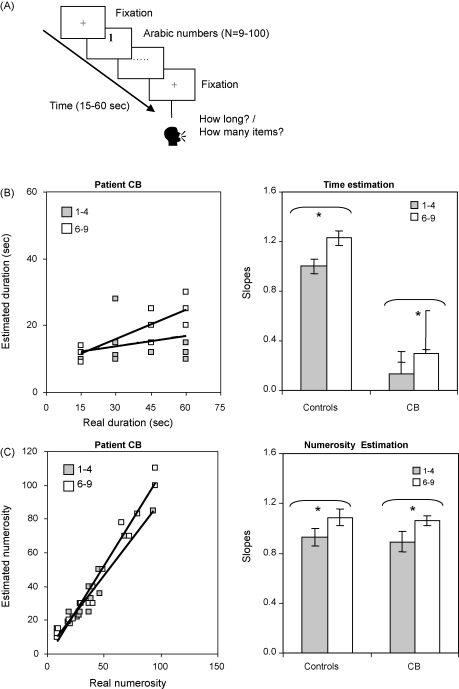
Task 3: Time and numerosity processing tested jointly. (A) Design of Tasks 3 and 4; (B) performance of patient CB and control subjects in time and (C) numerosity estimation of small (1–4) and large (6–9) numerical stimuli. Estimated durations (in s) and numerosities (number of items) are expressed as a function of real duration and numerosity respectively (patient CB, left panels) and as slopes (right panels) with 95% confidence limits for patient CB and control subjects.

There were three experimental tasks. The first two tasks (Tasks 3 and 4) used an identical experimental design as the time and numerosity estimation tasks (see Tasks 1 and 2). The only difference was in the stimuli used. Arabic numerals ranging from 1 to 9 (except 5) were presented instead of circles (Fig. 3A). In two different sessions, participants were asked to estimate either the duration of a trial or the number of Arabic numerals contained in each trial. In order to test whether the size of the numbers influenced time or numerosity estimation, 2 sets of numerical stimuli were used: small numbers (1–4) and large numbers (6–9). Moreover, in order to test whether there was any effect of temporal discrimination on numerical information we used two stimulus durations: fast (200–1100 ms) and slow (1101–2000 ms). Trials containing either small or large numbers of either slow or fast duration were randomly presented in equal proportion in 4 blocks of 16 trials each (total = 64). In 2 blocks participants were asked to estimate the duration of the trial (time estimation). In the other two blocks subjects were asked to estimate the number of stimuli presented (numerosity estimation). The order of the blocks was counterbalanced across subjects. During each trial, subjects were required to read the numbers aloud to prevent sub-vocal counting and to divert attention from timing. Similar to the tasks with coloured circles, the fast presentation of the numerical stimuli (i.e. 200–1100 ms for half of the trials) was designed to further prevent any sub-vocal counting. In both the time and the numerosity estimation tasks with numerical stimuli there was no time constraint to produce an answer. Responses were recorded and scored by the experimenter.

Since a number of studies have suggested that time intervals tend to be underestimated when subjects perform a concurrent task (e.g. Casini, Macar, & Grondin, 1992; Grondin & Macar, 1992; Harton, 1938; Macar, Grondin, & Casini, 1994; Zakay, 1993; see Thomas & Cantor (1978) for an exception), we used an additional ‘Time Discrimination’ task (Task 5). This was adapted from an existing paradigm (Oliveri et al., 2008) in order to evaluate time estimation without a concurrent task. Participants were asked to compare the duration of two numerical stimuli. Stimuli consisted of sequential pairs of centrally presented Arabic numbers subtending 1.72° of visual angle. Trials began with a central fixation point that remained visible until a key press from the participants. The first number then appeared in the middle of the screen for 600 ms followed by an ISI of 200 ms and by the second number. The first number was a 600 ms fixed-duration reference number (‘5’) while the second number could be either ‘1’, ‘5’ or ‘9’, and was either of a shorter or longer duration relative to the reference. A range of durations between 360 and 840 ms was used in steps of 60 ms. These durations were chosen on the basis of previous studies with neurological patients (e.g. Harrington et al., 2004). There were 4 shorter and 4 longer durations presented in equal proportion in 45 trials. At the end of each trial (reference, ISI and target stimulus), a white question mark presented in the position of the stimulus prompted participants to make a judgment. Subjects were required to indicate whether the second number lasted longer or shorter than the first number using the left (for shorter) or right arrow (for longer) keys on the laptop keyboard. Only these three target numbers rather than the whole range from 1 to 9 was used. This is because we aimed to keep the task's overall length adequate to be administered to the patient while maximizing the chances to observe any effect of numerical magnitude on time perception. Therefore, only numbers at the extreme of the single-digit range, i.e. the smallest and the largest were used.

#### 4.3.1 Results: time and numbers

Patient CB underestimated the duration of temporal intervals when Arabic numbers were used while performing a secondary task, i.e. reading Arabic numbers aloud [slope = 0.21, SE = 0.05 significantly different from 1, *p* < 0.05, Fig. 3B]. His performance differed significantly from control subjects [Satterthwaite's test (SingSlope, Crawford & Howell's test (Test a)): *t*(6) = −2.9, *p* < 0.01], even when CB was compared to the subgroup of controls that performed all experimental tasks [Satterthwaite's test (SingSlope, Crawford & Howell's test (Test d.1)): *t*(4) = −1.5, *p* = 0.035].

This impairment was not simply due to the fact that CB performed a concurrent task. Indeed, he was impaired at discriminating between temporal intervals in the time discrimination task (Task 5), which did not involve a concurrent task. In this task, he reached only 61% correct answers for stimuli duration differing by 150 ms from the reference stimulus (not significantly different from 50% chance level performance, Binomial probability *p* = 0.17), and performed at chance with shorter stimulus durations.

In contrast, his ability to judge the number of Arabic stimuli presented in an interval was intact (slope = 1.02, SE = 0.04, not significantly different from 1, Fig. 3C). His performance did not differ significantly from control subjects [Aspin's test (SingSlope, Crawford & Howell's test (Test b)): *t*(28) = 0.147, *p* = 0.44, n.s.], even when CB was compared to the subgroup of controls that performed all experimental tasks [Aspin's test (SingSlope, Crawford & Howell's test (Test b)): *t*(9) = 2.77, *p* = 0.3, n.s.].

Control subjects showed no significant impairment estimating either time [slope = 0.96, SE = 0.08, not significantly different from 1, Fig. 3B] or numerosity when Arabic numbers were used [slope = 0.97, SE = 0.01 not significantly different from 1, Fig. 3C]. Their performance in the time discrimination task was also intact, i.e. they reached ceiling performance (100% correct) for stimuli differing in duration by 150 ms from the reference stimulus.

#### 4.3.2 Interactions

##### 4.3.2.1 Stimuli size and duration on time processing

*Time estimation* (*Task* 3): Despite being impaired, CBs’ time estimation was modulated by the quantity expressed by numbers [Kruskal–Wallis main effect of stimulus size, *χ*^2^ = 2.145, *p < *0.003, see Fig. 3B]. Strikingly, smaller numbers (i.e. 1–4) resulted in more marked underestimation of time [slope = 0.11, SE = 0.07] relative to bigger numbers [i.e. 6–9, slope = 0.29, SE = 0.01].

As for Task 1, we tested whether performance was influenced by the duration of the individual stimuli. This analysis showed that the patient's time estimation was not influenced by the duration of the individual stimuli [Kruskal–Wallis main effect of stimulus duration, χ^2^ = −1.07, *p = *0.3, n.s.]. Therefore, stimuli of short durations did not result in perceiving intervals as lasting shorter [slope = 0.58, SE = 0.071], or stimuli of long durations in perceiving intervals as lasting longer [slope = 0.61, SE = 0.04].

Separate regression analyses were performed for each control subject to derive individual slope estimates for small and large numbers, which were then entered into an ANOVA. This showed that control subjects’ time estimation was influenced by the quantity expressed by numbers [*F*(1, 7) = 12.19, *p* *<* 0.01, Fig. 3B]. Therefore, big numbers (i.e., 6–9) resulted in perceiving longer durations [i.e., 6–9, slope = 1.23, SE = 0.18] relative to small numbers [i.e., 1–4, slope = 1.00, SE = 0.2]. There was also a trend for a significant effect of the stimuli duration of the perceived overall duration, such that stimuli lasting longer tended to make the overall interval being perceived as lasting longer [*F*(1, 7) = 5.56, *p* *=* 0.051].

The effect of stimulus duration on time estimation did not differ between CB and the control group for both short [SingSlope, Crawford & Howell's test (Test c): *t*(7) = 0.13, *p* = 0.44, n.s.] and long durations [SingSlope, Crawford & Howell's test (Test c): *t*(7) = 0.67, *p* = 0.25, n.s.]. This difference was not significant even when the patient's performance was compared to the subgroup of controls that performed all experimental tasks [short durations: SingSlope, Crawford & Howell's test (Test c): *t*(5) = −0.23, *p* = 0.3, n.s.; long durations: SingSlope, Crawford & Howell's test (Test c): *t*(5) = 0.12, *p* = 0.48, n.s.].

In contrast, CBs underestimation of temporal durations was even more dramatic relative to control subjects for both small [SingSlope, Crawford & Howell's test (Test c): *t*(7) = 5.32, *p* < 0.01] and large number values [SingSlope, Crawford & Howell's test (Test c): *t*(7) = 3.08, *p* < 0.04], even with compared with the controls’ subgroup [small number values: SingSlope, Crawford & Howell's test (Test c): *t*(5) = 8.11, *p* < 0.001] and large number values [SingSlope, Crawford & Howell's test (Test c): *t*(5) = 4.3, *p* < 0.03].

*Time discrimination* (*Task* 5): An analysis on the values indicating proportion of test ‘longer’ responses for each number indicated that CBs’ performance was not modulated by the quantity expressed by numbers stimuli [Kruskal–Wallis main effect of number quantity, *χ*^2^ = 5.01, *p* *=* 0.08, n.s.].

In contrast, control subjects’ ability to discriminate between temporal durations was modulated by the quantity expressed by numbers [*F*(2, 8) = 7.17, *p* *=* 0.017]. The results of fitting PSEs to the control data revealed that relative to the test value ‘5’, the duration of the test value of ‘9’ had to be 44 ms shorter (*t*(4) = −3.50, *p* *<* 0.02) in order to be perceived as equal to the reference stimulus ‘5’ whereas for the test value ‘1’ had to be only 5 ms shorter [*t*(4) = −0.33, *p* *=* 0.76, n.s.], see Appendix 1.

##### 4.3.2.2 Stimuli size and duration on numerosity estimation

CBs’ numerosity estimation was influenced by the quantity expressed by numbers [Kruskal–Wallis main effect of stimuli size, *χ*^2^ = 4.14, *p* *<* 0.01]. Therefore, smaller numbers resulted in the interval being perceived as containing fewer stimuli [slope = 0.99, SE = 0.05] and larger numbers in the interval being perceived as containing more stimuli [slope = 1.1, SE = 0.07, Fig. 3C]. In contrast, the patient's numerosity estimation was not influenced by the stimuli duration [Kruskal–Wallis main effect of stimuli duration, *χ*^2^ = 1.02, *p* *=* 0.35, n.s.].

Separate regression analyses were performed for each control subject to derive individual slope estimates for small and large numbers, which were then entered into an ANOVA. This showed that control subjects’ numerosity estimation was influenced by the quantity expressed by numbers [*F*(1, 11) = 21.7, *p* *<* 0.001]. Therefore, smaller numbers resulted in the interval being perceived as containing fewer stimuli [slope = 0.90, SE = 0.04] and larger numbers in the interval being perceived as containing more stimuli (slope = 1.06, SE = 0.09, see Fig. 3C). In contrast, controls’ numerosity estimation was not influenced by the stimuli duration [*F*(1, 11) = 3.48, *p* *=* 0.4, n.s.].

The effect of stimuli duration on numerosity estimation did not differ between CB and the control group [short duration: SingSlope, Crawford & Howell's test (Test c): *t*(11) = 0.13, *p* = 0.44, n.s.; long duration: SingSlope, Crawford & Howell's test (Test c): *t*(11) = 0.29, *p* = 0.7, n.s.]. This difference was also not significant when CB was compared to the subgroup of controls that performed all experimental tasks [short duration: SingSlope, Crawford & Howell's test (Test c): *t*(5) = 0.8, *p* = 0.52, n.s.; long duration: SingSlope, Crawford & Howell's test (Test c): *t*(5) = 0.35, *p* = 0.67, n.s.].

Likewise, the effect of stimuli size on numerosity estimation did not differ between CB and both the overall control group and the control subgroup [Overall group: small size: SingSlope, Crawford & Howell's test (Test c): *t*(11) = 0.67, *p* < 0.25, n.s.; large size: SingSlope, Crawford & Howell's test (Test c): t(11) = 0.13, *p* = 0.44, n.s. Subgroup: Small size: SingSlope, Crawford & Howell's test (Test c): *t*(5) = 0.67, *p* *=* 0.25, n.s.; large size: SingSlope, Crawford & Howell's test (Test c): *t*(5) = 0.41, *p* = 0.2, n.s.].

##### 4.3.2.3 Interim discussion

These results showed that patient CB was selectively impaired at estimating time and that his performance was nevertheless modulated by the quantity expressed by numbers. In contrast, his ability to estimate numerosity was intact, and also modulated by number quantity. We note that tasks assessing time and numerosity estimation were identical in terms of the stimuli and the experimental procedure used, apart from the actual task performed. Therefore, it is unlikely that any difference in performance between these tasks could simply be attributed to differences in terms of perceptual or attentional resources required to perform the tasks. Control subjects were accurate in time and numerosity estimation, as well as in the time discrimination task. In these tasks their performance was modulated by the quantity expressed by numbers.

### 4.4 Section 4. Space processing

A ‘Line Length Discrimination’ (Task 6) was used to test space processing. Participants had to indicate which of two lines was the longer. Trials began with a central fixation point that remained visible until a key press from the participants. After a blank interval of 200 ms a reference and a target line were presented one 5.07° above the horizontal meridian and the other 5.07° below, in randomized order. The lines appeared centred on the vertical meridian one after the other for 600 ms separated by an inter-stimulus interval of 100 ms. The reference line subtended 5.15° of visual angle in length (∼4.5 cm); target lines varied in length relative to the reference by ±0.26 or 0.52° of visual angle (∼±0.23 or 0.45 cm). Participants indicated the line with the longer length by depressing the upper or the lower arrow key on the computer keyboard.

Note that the difference between the number of stimulus levels in this task (four) versus the time discrimination (Task 5) (eight) should not affect the measure of interest, namely just noticeable differences (JNDs, see Section 4) which are likely to remain the same regardless of the number of stimulus levels used to find it. As the aim of this experiment was to probe spatial processing in general, we did not attempt to monitor ocular-motor behaviour to distinguish between spatial processes that may be dependent or independent of eye movements. Moreover, we were not motivated to monitor eye gaze in CB in particular, as no deficits in eye gaze or eye movements had been reported in his medical history.

#### 4.4.1 Results: space processing

To establish to what extent CBs’ performance in the Line Length Discrimination Task (Task 6) differed from control subjects, CBs’ just noticeable distance (JND) was expressed as a *z*-score relative to the controls’ JNDs. The controls’ JNDs had a mean (and standard deviation) of 3.54 (1.17). CBs’ JND was 5.84, and his *z*-score was therefore 1.56. This result suggests that the patient's performance was within the normal range, i.e. that the probability of the patient's JND coming from same population as the controls’ was greater than *p* = 0.05. A direct comparison of CBs’ and control subjects’ performance (JNDs) also confirmed that these did not differ [ST: *t*(7) = 1.85, *p* = 0.11, n.s.] even when CB was compared to the control subgroup [ST: *t*(5) = 0.46, *p* = 0.33, n.s.].

### 4.5 Section 5. Space and numbers

Two tasks were used to test space and number processes together. The first task (Task 7) was based on a classical experimental paradigm used to reveal implicit spatial–numerical associations (known as the SNARC effect, Dehaene et al., 1993). Subjects initiated each block by pressing the spacebar. Each trial began with a central fixation cross for 200 ms, after which Arabic numbers from 1 to 9 (excluding 5) were centrally presented one at the time for 200 ms. There were two blocks of 128 stimuli each, thus each Arabic number was presented 32 times. Participants had to decide whether each of the numbers presented was odd or even, i.e. parity judgment task. Stimuli remained on the screen until participants made an answer or for a maximum of 1500 ms. Following a procedure commonly used when administering this task (Dehaene et al., 1993), participants responded by depressing either the left (odd numbers) or the right (even numbers) arrow keys of the keyboard. The assignment of the ‘odd’ and ‘even’ responses to the left and right key was reversed in the two blocks and the order of the instructions was counterbalanced across subjects.

The second task (Task 8) was number comparison, where participants had to decide whether a number was larger or smaller than the reference ‘5’. Each block started with the word ‘Experimental Task’ that remained in the middle of the screen until subjects pressed the spacebar. This was followed by a central fixation cross presented for 200 ms after which Arabic numbers from 1 to 9 (excluding 5) were presented one at the time for 200 ms. There were 2 blocks of 128 single-digit Arabic numbers each. Numbers appeared at a visual angle of 4.89° either to the left or the right of the fixation point on the computer monitor, following a procedure previously used in other studies (e.g. Lavidor, Brinksman, & Göbel, 2004). Participants had to decide whether each number was larger or smaller than 5. In one block, they were requested to press a left key of the keyboard if the number presented was smaller than 5 and a right key if the number was larger than 5. The instructions were reversed for the following block and the order of the instructions was counterbalanced across participants. The lateralized presentation of the numerical stimuli aimed to test whether there was any interaction of space on numerical processing, i.e. whether judgments of number magnitude could be modulated by the spatial location of the numbers on the screen. On the basis of the hypothesis that numbers are represented along a left-to-right oriented mental line (Dehaene et al., 1993), participants’ response times in both tasks were expected to be faster when small numbers were answered with the left hand and when large numbers were answered with the right hand.

#### 4.5.1 Results: space and numbers

Patient CB was accurate at judging the parity of numbers (Task 7) as well as at comparing their magnitude (Task 8) (91.4% and 98% correct answers, respectively). In the number comparison task, the patient's response times decreased as a function of the numerical distance from number 5 [*r*^2^ = 0.128, *p* *<* 0.001], therefore indicating a normal distance effect (Moyer & Landauer, 1967).

Control subjects were accurate at indicating the parity status of numbers (96.8% correct answers) and the larger between two numbers (98.7% correct answers). Their performance in magnitude comparison was modulated by the numerical distance from the reference number ‘5’ [*r*^2^ = 0.58, *p* *<* 0.006]. Patient CBs’ accuracy did not significantly differ from the overall control group and from the controls’ subgroup in either tasks [overall control group: parity judgment: ST: *t*(7) = −0.24, *p* = 0.75, n.s.; magnitude ST: *t*(7) = −0.31, *p* = 0.76, n.s.; controls subgroup: parity ST: *t*(5) = −0.89, *p* = 0.21, n.s.; magnitude ST: *t*(5) = −0.66, *p* = 0.27, n.s.]. However, CBs response times were significantly slower than controls in both tasks [parity judgment: mean RTs control subjects: 419.3 ms, patient CB: 1242.14 ms; ST: *t*(7) = 8.670, *p* *<* 0.001; magnitude comparison: mean RTs control subjects: 461.3 ms, patient CB: 643.8 ms; ST: *t*(7) = 13.42, *p* *<* 0.001]. This was also the case when the patient was compared to the controls’ subgroup in both tasks [parity judgment: *t*(5) = 21.9, *p* *<* 0.001; magnitude comparison: ST: *t*(5) = 4.65, *p* *<* 0.002].

#### 4.5.2 Interactions

##### 4.5.2.1 Numbers on space processing

In the parity judgment task, the slope of the linear regression between [right–left] key presses and each number presented was calculated for each participant. In patient CB this analysis showed a superiority of the left hand to answer small numbers and of the right hand to answer large numbers [*r*^2^ = 0.167, *p* *<* 0.03]. Therefore, large numbers on the right were answered about 22 ms faster than on the left, and the opposite was true for small numbers (i.e. 18 ms difference between left and right). This is consistent with the SNARC effect and suggests that CBs spatial representation of numbers along a mental line is preserved (Dehaene et al., 1993).

Similarly, control subjects were faster at processing small numbers with the left hand and large numbers with the right hand [*r*^2^ = 0.68, *p* *<* 0.001]. These results suggest an interaction of number over space processing in both patient CB and control subjects, with no difference between them [overall control group: RSDT: *t*(7) = −0.33, *p* = 0.37, n.s.; controls’ subgroup: RSDT: *t*(5) = 0.69, *p *= 0.25, n.s.].

##### 4.5.2.2 Space on number processing

In the number comparison task, CBs’ ability to judge the magnitude of numbers was modulated by their location on the computer monitor [Kruskal–Wallis main effect of space, *χ*^2^ = 8.68, *p* *<* 0.03]. Specifically, small numbers were judged faster when presented on the left side of the monitor [Mann–Whitney *U*, *χ*^2^ = −2.93, *p* *<* 0.03,] and large numbers when presented on the right side of the monitor [Mann–Whitney *U*, χ^2^ = −6.32, *p* *<* 0.001].

Control subjects’ performance was also modulated by the location of the stimuli on the monitor [*F*(1, 7) = 7.93, *p* *<* 0.02], such that they were faster at judging the magnitude of small numbers when presented on the left side of the monitor [*t*(7) = 2.41, *p* = 0.034] and of large numbers when presented on the right side [*t*(7) = 2.5, *p* *<* 0.030]. Consistent with previous studies using a similar experimental paradigm (e.g. Lavidor et al., 2004), these results suggest an interaction of space over number processing in both patient CB and control subjects, with no difference between them [overall control group: RSDT: *t*(7) = −0.146, *p* = 0.44, n.s.; controls’ subgroup: RSDT: *t*(5) = 1.1, *p *= 0.16, n.s.].

## 5 Discussion

In this study, we undertook for the first time a detailed investigation of time, number and space processing in a patient with a right hemisphere lesion.

### 5.1 A selective impairment in time processing

Our results indicated that patient CB was selectively impaired in processing time. In contrast, number and space processing were preserved. CBs’ impairment in time processing encompassed both the ability to estimate temporal intervals as well as the ability to discriminate between temporal durations. CBs’ underestimation of temporal intervals could be up to 1/3 of their veridical duration (e.g. 18 s for a veridical 60 s interval) and did not depend on whether the stimuli were coloured circles or Arabic numbers. CBs’ underestimation of temporal intervals in the ‘time estimation’ task (Task 1) occurred while performing a concurrent task (naming colours or reading numbers aloud). It has been reported that even healthy subjects underestimate time when performing a concurrent task (e.g. Casini & Macar, 1997; Gibbons & Rammsayer, 2004), and indeed our control subjects showed a similar effect. Therefore it could be argued that CBs’ underestimation may simply be due to a response-bias in dual-task performance. However, two aspects seem to rule out this possibility. The first is the effect of numerical value on time perception, i.e. a sequence of small numbers was perceived as lasting shorter and a sequence of large numbers as lasting longer than their veridical duration. The second aspect suggesting that underestimations could not simply be explained by response bias in dual-task is that CB was also impaired in the ‘time discrimination task’ where he was asked to judge which of two stimuli lasted longer without performing any concurrent task.

It is interesting to note that CBs’ errors in temporal tasks always consisted of underestimations. Impaired time processing may be expected to result in random errors (i.e. a mixture of accurate responses, under and over-estimations) rather than systematic tendency in one direction, i.e. underestimations in this case. However, we note that a similar tendency to underestimate temporal intervals has been shown in some previous neuropsychological patients (e.g. Danckert et al., 2007; Drane et al., 1999; Meck, 2005; Richards, 1973; Vidalaki et al., 1999). Temporal underestimations have been explained in terms of the inefficiency of some components involved in time processing and in particular of the pacemaker component, although an isolated deficit to this component does not seem sufficient to cause time impairment (Danckert et al., 2007). At present it is not possible to specify which component involved in time processing was at fault in patient CB.

It might also be argued that time estimation was influenced by the number of stimuli presented. Indeed, in the ‘time estimation’ task (Task 1) there was a positive correlation between the number of stimuli presented and the total duration of the sequence. Thus a subject who either knew the frequency of the stimuli or received reliable feedback on their responses would be able to correctly report the duration of the sequence based on simply counting the number of items seen. However, such a strategy would have been difficult in the present paradigm as feedback was not given, and the frequency of stimuli was both randomized between sequences and varied randomly within sequences along a wide continuous range (e.g. within ‘fast’ and ‘slow’, as described above in Section 4.1), while continuous verbal output and fast presentations should have impaired counting of the stimuli. Despite such factors interfering with formation of learned associations based on correlation, control subjects could perform the task with near-veridical accuracy.

In striking contrast with his time impairment, CBs’ number and space processing were spared. In a series of paper-and-pencil tasks, the patient showed good performance in estimating the numerosity of a set or the area covered by objects. He could also correctly perform arithmetical operations and number comparison. Moreover, the patient performed well when asked to estimate the numerosity of the same sets of stimuli previously used in ‘time estimation’ task, regardless as to whether the stimuli consisted of coloured circles or Arabic numbers. Spatial processing was also entirely preserved in patient CB. In three stringent tests used to assess neglect, CBs’ performance was normal and he also performed well in the ‘line length discrimination’ task (Task 6), requiring to indicate the longer of two lines.^4^

It might be suggested that CBs selective impairment in time estimation was simply due to time tasks being more difficult than the tasks used to test numerosity and spatial estimation. Although we cannot completely rule out differences in task difficulty, we tried to avoid this by equating the critical tasks on as many parameters as possible. As explained in the Methods, the tasks assessing time and numerosity estimation (Tasks 1–4) were identical in terms of the experimental stimuli and procedure used, the only difference being in the instructions given to the participants. Moreover, although we could not equate the line length discrimination task (Task 6) to the other tasks in a similar way, we note that the line length discrimination task is unlikely to suffer from task difficulty issues. This is because what we measured is the threshold at which subjects were able to detect differences between two stimuli. Specifically, we tested participants’ ability across several difficulty levels ranging from easy to hard to find the ‘just noticeable difference’ (JND) level at 75% correct. It should arguably be similarly difficult to detect a JND in line length as it is to detect a JND in stimuli duration regardless of how many levels are used to find it.

Our data provide a clear single dissociation in patient CB between impaired time processing on the one hand, and spared number and space processing on the other. A double dissociation among these magnitude dimensions would indeed further advance our understanding of the relation between them. Such dissociation has yet to be reported in patients with neurological lesions. However, the relation between time and number processing has recently been examined in a group of six adults with developmental dyscalculia (DD), an innate impairment in understanding and manipulating numbers (e.g. Butterworth, 2005). By using a variant of the experimental tasks employed to study patient CB, our preliminary data show that time processing in DD is preserved despite numbers being impaired (Cappelletti, Freeman, & Butterworth, forthcoming). This therefore suggests that a double dissociation seems to exist at least between time and numbers.

### 5.2 Interactions between time, numbers and space

We investigated whether interactions between numbers and time and between numbers and space occurred. We found a significant interaction between numbers and time. Thus, small numbers (1–4) made the time intervals be perceived as shorter than its veridical duration, and large numbers (6–9) as longer than its veridical duration. Interestingly, the effect was present in both patient CB and in control subjects, the only difference being that CBs’ underestimation was even more dramatic that in controls. This interaction of numerical quantity and time has not previously been reported in neuropsychological patients and suggests a connection between number and time processing. Numerical quantity also interacted with the numerosity of the set (i.e. how many items), such that small numbers made the interval be perceived as containing fewer stimuli than the veridical amount and large numbers as containing more than the veridical amount.

Patient CB and control subjects’ estimation of time and numerosity was not modulated by the duration of the stimuli, with the exception of a trend in time estimation in control subjects. Therefore stimuli of short duration did not make an interval be perceived as shorter than its veridical duration or containing fewer stimuli. Similarly, stimuli of long duration did not make an interval be perceived as lasting longer than its veridical duration or containing more stimuli. A similar unidirectional interaction between numbers and time has already been reported in studies based on the Stroop paradigm and on temporal bisection (Dormal et al., 2006, 2008; Droit-Volet, 2003). This has been explained as reflecting the fact that numerosity, but not time, is automatically accessed even when task-irrelevant (e.g. Dormal et al., 2006; Droit-Volet, 2003). This proposal found support in the several experiments showing the automatic access to numerosity, whether this consisted of symbolic or non-symbolic material (e.g. Dehaene & Akhavein, 1995; Koechlin, Naccache, Block, & Dehaene, 1999; Tzelgov, Meyer, & Henik, 1992). The prevalence of the numerical dimension over the temporal one may also be the consequence of the advanced use of numbers in the context of exact calculation (Walsh, 2003). It is also possible that judgments on numerosity are more frequent and explicit than those on duration (Dormal et al., 2006).

Our data do not allow us to draw strong conclusions on whether the unidirectional interaction we found is driven by the automatic access to numerical stimuli. We note, however, that besides the interaction between numbers and time, our data also showed a bidirectional interaction between numbers and space. An interaction of space on number processing was observed in both patient CB and control subjects’ judgment of number magnitude which was modulated by the spatial location of the numbers on the computer monitor. Moreover, interaction of numbers on space processing was observed in the parity judgment task, where subjects’ judgment of the parity status of numbers was modulated by their spatial location on the mental number line, i.e. small on the left and large on the right. This interaction between space and numbers is well-documented in both healthy controls and neurological patients (e.g. Cappelletti & Cipolotti, 2006; Dehaene et al., 1993; Doricchi et al., 2005; Hubbard et al., 2005; Zorzi et al., 2002). The effect of space on numbers therefore suggests that not all interactions can be due to a stronger effect of numbers relative to other magnitude codes.

Our study did not directly explore the relation between time and space because of time constraints. This relation has been investigated by some recent studies which provided evidence of strong interactions between these two dimensions (e.g. Casasanto & Boroditsky, 2008; Ishihara, Keller, Rossetti, & Prinz, 2008; Vallesi, Binns, & Shallice, 2008). For instance, it has been shown that subjects observing differently scaled environments undergo systematic shifts in the experience of time, such that temporal durations are perceived as shorter in the presence of small space (De Long, 1981). Moreover, a recent study reported that subjects performing time comparisons showed faster left-side responses to early onset timings and faster right-side responses to late onset timings (Ishihara et al., 2008). A link between time and space has also been indicated by neuropsychological studies reporting the co-occurrence of spatial and temporal deficits in patients with neglect (Basso, Nichelli, Frassinetti, & di Pellegrino, 1996; Danckert et al., 2007). It may be possible that the interaction between time and space is not completely symmetrical, that is time may exert a greater influenced on space than the opposite. Although some existing studies have shown that time also influences space processing to some extent (e.g. Ishihara et al., 2008; Vallesi et al., 2008), no studies have yet examined the mutual interaction of time and space within the same experimental paradigm.

Although the relation between time and space was not directly investigated in this study, our evidence of the dissociation between impaired time processing and preserved numerical and spatial processing seems sufficient to claim the partial independence among these three dimensions. Future studies are needed to complement this evidence by directly assessing the relation between time and space in the context of numerical processing as well.

### 5.3 Implications for single or multiple magnitude systems

Our findings of a selective impairment in time processing allow us to evaluate the two main contrasting proposals suggesting that time, numbers and space are implemented by either distinct magnitude systems or by a common one. The proposal of distinct magnitude systems can easily accommodate CBs selective impairment for time. According to such proposal, distinct magnitude systems underpin distinct magnitude dimensions. This leads to the prediction that one magnitude system can be impaired while the others continue to work normally. However, we found that number quantity modulated the participants’ perception of time even in patient CB despite his time impairment. This interaction is problematic for such position since it was not expected if these magnitude dimensions are fully independent.

Our data are also problematic for proposals suggesting that a fully shared accumulator mechanism underpins time and number processing (e.g. Meck & Church, 1983). First, we have shown that number processing (as well as space) was normal in patient CB, despite his severe impairment in time processing. This is difficult to account for because a fully shared mechanism would have resulted also in an impairment in number processing.

We suggest that our data are best explained by assuming that time, numbers and space only partly share a common system. Two aspects of these results are critical. First, they allow us to clarify to what extent different magnitude dimensions are independent. In particular, CBs’ selectively impaired time processing showed that this is distinct from numbers and space. An ideal experimental design would combine time, numbers and space in a single experiment in order to test systematic co-variations in magnitude judgments across these domains. Although we could not run such experiment on patient CB, our results nevertheless suggest that time, numbers and space are partly independent as they can be selectively impaired and yet they can modulate each other as shown by their interactions.

Second, our data allow us to suggest what is in common between different magnitude dimensions. Two possibilities exist: one is that these dimensions are represented by a shared magnitude system. However, this system is unlikely to be based on an accumulator principle such as proposed by previous authors (e.g. Meck & Church, 1983). This is because an accumulator principle cannot account for processing space as this dimension is not represented through events or objects to be accumulated in the same way as numbers and time do. The other possibility, which differs from the ATOM (Walsh, 2003), is that different magnitude dimensions share the same operational mechanisms, for instance those used to perform comparisons. Common operational mechanisms have been previously hypothesized to account for infants’ different performance with numerosities and continuous magnitudes (Huntley-Fenner, Carey, & Solimando, 2002; McCrink & Wynn, 2004). Some studies have shown that infants react to changes in numerosity (McCrink & Wynn, 2004) but not to changes in continuous magnitude (Huntley-Fenner et al., 2002). This result has been explained in terms of a shared comparison mechanism that operates on different magnitude dimensions rather than in terms of a shared magnitude system. Moreover, the idea that magnitude dimensions may be sharing the same operational mechanisms, for instance comparison mechanisms, found some support in studies investigating whether processing numbers and other magnitude dimensions activate same or different responses (Cohen Kadosh et al., 2007). For instance, it has been shown that numbers and physical size are processed separately until response-related stages as indicated by an interaction of these dimensions in the primary motor cortex (Cohen Kadosh et al., 2007). At a speculative level it can be suggested that shared mechanisms may be more compatible with the nature of different magnitude dimensions rather than a shared representational format. Our data on space and number interaction would be consistent with the hypothesis of shared (comparison) mechanisms although they do not allow us to further speculate on these two options.

### 5.4 Time, numbers and space in the brain

What are the anatomical bases of time, number and space processing? It has been proposed that the locus of the common magnitude system is the right inferior parietal cortex (e.g. Walsh, 2003). Indeed this area, among others, is often damaged in patients with impaired time perception (Harrington et al., 2004; Koch et al., 2003; Meck, 2005). It has also been reported in several imaging studies (e.g. Lejeune et al., 1997; Maquet et al., 1996; Rao et al., 2001), although time impairments also arise from lesions in the cerebellum (for the milliseconds range, e.g. Ivry & Spencer, 2004) and in the basal ganglia (for seconds range, e.g. Matelli & Meck, 2000, 2004; Meck & Benson, 2002). Moreover, the right inferior parietal lobe, together with its homologous on the left hemisphere, has been identified as a key brain region for processing numbers (e.g. Dehaene et al., 2003) and numerical impairments often follow left hemisphere damages (e.g. Cipolotti et al., 1991; Cipolotti & van Harskamp, 2001; Dehaene & Cohen, 1997; Lemer et al., 2003). Finally, it has been shown that space processes involve the parietal areas and indeed impairments to these processes such as neglect to either physical or numerical space are often due to lesions in the right parietal lobe (e.g. Driver & Vuilleumier, 2001; Doricchi et al., 2005^5^; Zorzi et al., 2002), although dissociations within spatial processing have also been reported, for instance between near and far space (e.g. Cowey, Small, & Ellis, 1994; Halligan & Marshall, 1991). Patient CBs’ brain lesion involved the right parietal lobes as well as several other regions in the right hemisphere. Therefore, our anatomical data do not allow us to draw any firm conclusion regarding the involvement of the parietal areas in time, numbers and space processing. We note, however, that despite this lesion number and space processing were still well preserved in CB. This is difficult to accommodate within the proposal of the ATOM that the common magnitude system would be located in the right parietal lobe (Walsh, 2003). Rather, our data allow us to make two suggestions. First, it is possible that besides the parietal areas, other brain regions are involved in processing the specific magnitude codes. This would explain why some of these codes are impaired and others are intact, in our case time relative to numbers and space. Support to this hypothesis comes from other studies showing that some parietal regions, and specifically the IPS, are only critical for numerical but not time processing (Dormal et al., 2008). This would be consistent with CBs’ brain lesion that spared the IPS areas. Secondly, it is possible that the neuronal correlates of different magnitude codes may recover in different ways following a brain lesion, revealing alternative neuronal and cognitive mechanisms for performing the tasks, i.e. degenerate systems (Price & Friston, 2002), or that the perilesioned areas are still sufficient for processing some of these dimensions but not others (e.g. Price & Friston, 2002). Although our data do not allow us to further speculate on this point, we note that degeneracy and recovery may account for inconsistencies between our results of a dissociation in performing different magnitude codes and other studies suggesting that the same parietal regions are equally activated when performing temporal and numerical tasks (e.g. Maquet et al., 1996; Pouthas et al., 2000).

In conclusion, the present study provides the first evidence that time processing can be selectively impaired while number and space processing is spared. Moreover, we showed that despite this impairment time processing was still modulated by number quantity. Our data are best accounted for by the hypothesis of a partly shared magnitude system or operational mechanisms between these dimensions. Although the right parietal regions appeared involved in time, numbers and space processing, there is clearly need to clarify their role in magnitude processing.

## Figures and Tables

**Figure:**
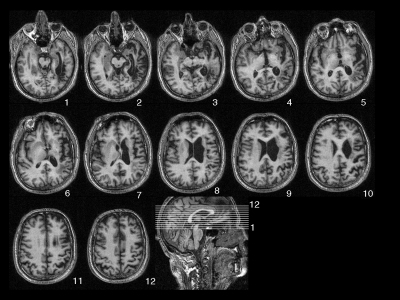


**Figure:**
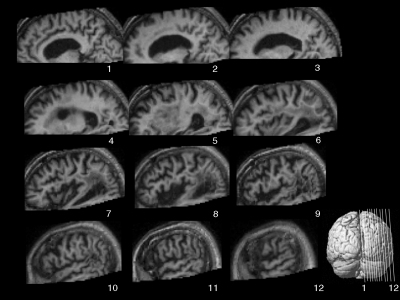


**Figure:**
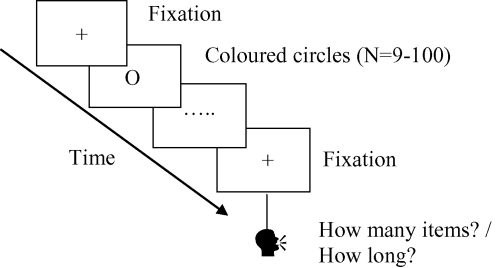


**Figure:**
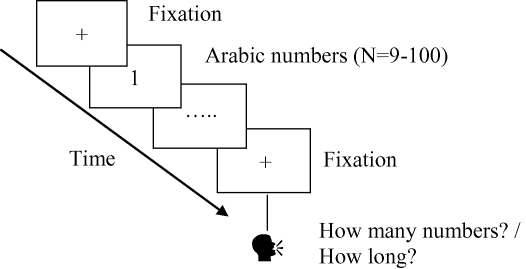


**Figure:**
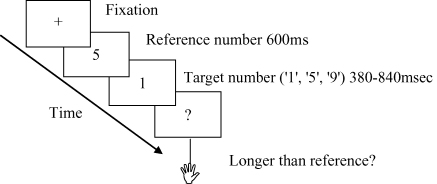


**Figure:**
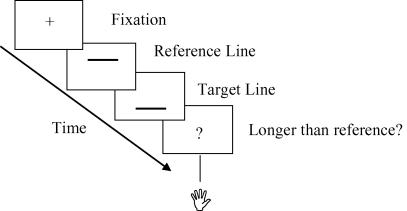


**Figure:**
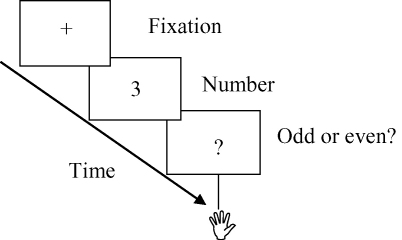


**Figure:**
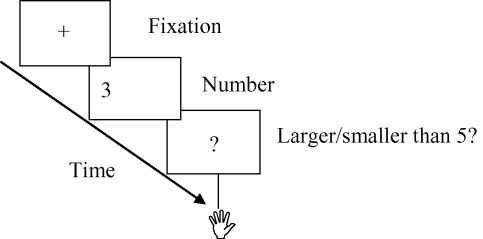


**Figure:**
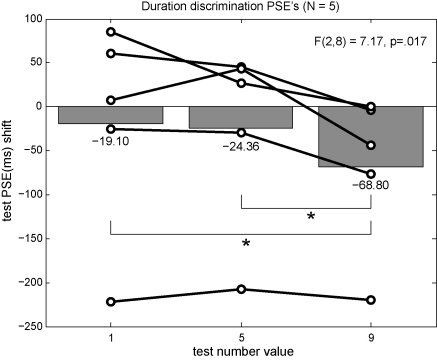


**Table 1:** Summary of patient CB and control subjects’ cognitive scores (number correct; percentiles or cut-off points in parenthesis).

Tasks performed	Patient CB
General intellectual functioning	
NART I.Q.	120
WAIS-R verbal I.Q.	113
WAIS-R performance I.Q.	91

Memory	
Recognition memory test	
Words	46/50 (>75th %ile)
Faces	45/50 (>75th %ile)

Word retrieval	
Graded difficulty naming test	27/30 (>75th %ile)

Executive functions	
WCST_No. categories	5/6
Hayling	6 (average)

Attention	
Elevator counting	7/7 (normal)
Elevator counting with distractors	9/10 (>75th %ile)

Perception	
Incomplete letters	20/20 (>5% cut-off)
Dot counting	10/10 (>5% cut-off)
Cube analysis	10/10 (>5% cut-off)

Neglect	
Balloon	Lateralized inattention index >45% (normal)
Bell crossing	15 right; 17 left
Line bisection	0.8 mm to the right

Calculation and number reading	
GDA test (*N* = 24)	10 (average)
Reading 1–4 Arabic numbers (*N* = 40)	98.7

NART = National Adult Reading Test; %ile = percentile; WCST = Wisconsin card sorting test; GDA = Graded Difficulty Arithmetic Test.

**Table 2:** Patient CB and control subjects’ performance in (A) time and (B) numeracy processing (number of correct responses and standard deviation in control subjects).

Tasks performed	Patient CB	Control subjects
A. Time processing		
Time estimation task (*N* = 30)	12	28.9 (3.5)
Knowledge of exact temporal facts (*N* = 20)	19	19.6 (2.3)
Time comparison task (*N* = 15)	14	14.7 (2.9)

B. Numeracy processing		
Numerical estimation task (*N* = 30)	24	28.3 (3.4)
Number comparison estimation (*N* = 24)	22	22.1 (3.2)
Calculation estimation (*N* = 18)	16	17.01 (4.2)
Area estimation (*N* = 52)	46	48.6 (4.6)
Number of squares estimation (*N* = 52)	44	48.1 (3.8)
Knowledge of exact number facts (*N* = 20)	19	20 (0)
Knowledge of arithmetical facts (*N* = 60)	57	56.4 (2.3)
Number comparison (*N* = 68)	68	68 (0)

## Appendix C Schematic representation of the design of the experimental tasks used

Tasks 1 and 2: Time and numerosity estimation with non-numerical stimuli in a concurrent task (naming circles’ colour).

Tasks 3 and 4: Time and numerosity estimation with numerical stimuli in a concurrent task (reading numbers aloud).

Task 5: Time discrimination with no concurrent task.

Task 6: Line length discrimination.

Task 7: Parity judgment.

Task 8: Number comparison.

## Footnotes

1 It may be claimed that the task assessing knowledge of temporal facts may not necessarily tap into time processing as it can potentially be answered with basic semantic knowledge, without having to compute the elapsed duration. However, whether it was the ability to compute time or the semantic knowledge referring to it, CBs performance in paper-and-pencil tasks suggests that his time processing was impaired.

2 We use the term ‘Time processing’ to refer all the types of manipulation of temporal information, therefore including ‘Time estimation’ and ‘Time discrimination’.

3 Although number and numerosity processing as well as the mechanisms for extracting sequential and simultaneous numerosity may engage different systems and are expected to lead to different computations, it was beyond the scope of the present study to investigate any differences in how such systems relate to time and space processing.

4 An anonymous reviewer suggested that the latter task might be solved by merely noting the position of the end-points of the lines. However, we note that in order to do so participants would have to engage in two distinct cognitive operations: first, noting the position of each of the line ends, and second to judge the orientation of an imaginary line connecting them (analogous to a Vernier task). Though we cannot discount this possibility a priori, this explanation does not seem as parsimonious as one based on comparing line lengths directly.

5 Note that Doricchi et al. (2005, 2009) have suggested that the right prefrontal areas are those more frequently associated with impairments to the ability to process numbers along a line. However, this does not mean that lesions to the parietal areas cannot cause such impairments. Rather, it means that these lesions are less frequently associated with number line impairments.
